# Supplementary material for: Cytotoxic CNS-associated T cells drive axon degeneration by targeting perturbed oligodendrocytes in PLP1 mutant mice
Source: iScience. 2023 Apr 19;26(5):106698. doi: 10.1016/j.isci.2023.106698 (PMC10172788; doi:10.1016/j.isci.2023.106698)
Supplement: Document S1. Figures S1–S6 [file mmc1.pdf]

iScience, Volume *26*

Supplemental information

Cytotoxic CNS-associated T cells drive axon degeneration by targeting perturbed oligodendrocytes in *PLP1* mutant mice

Tassnim Abdelwahab, David Stadler, Konrad Knöpper, Panagiota Arampatzi, Antoine-Emmanuel Saliba, Wolfgang Kastenmüller, Rudolf Martini, and Janos Groh

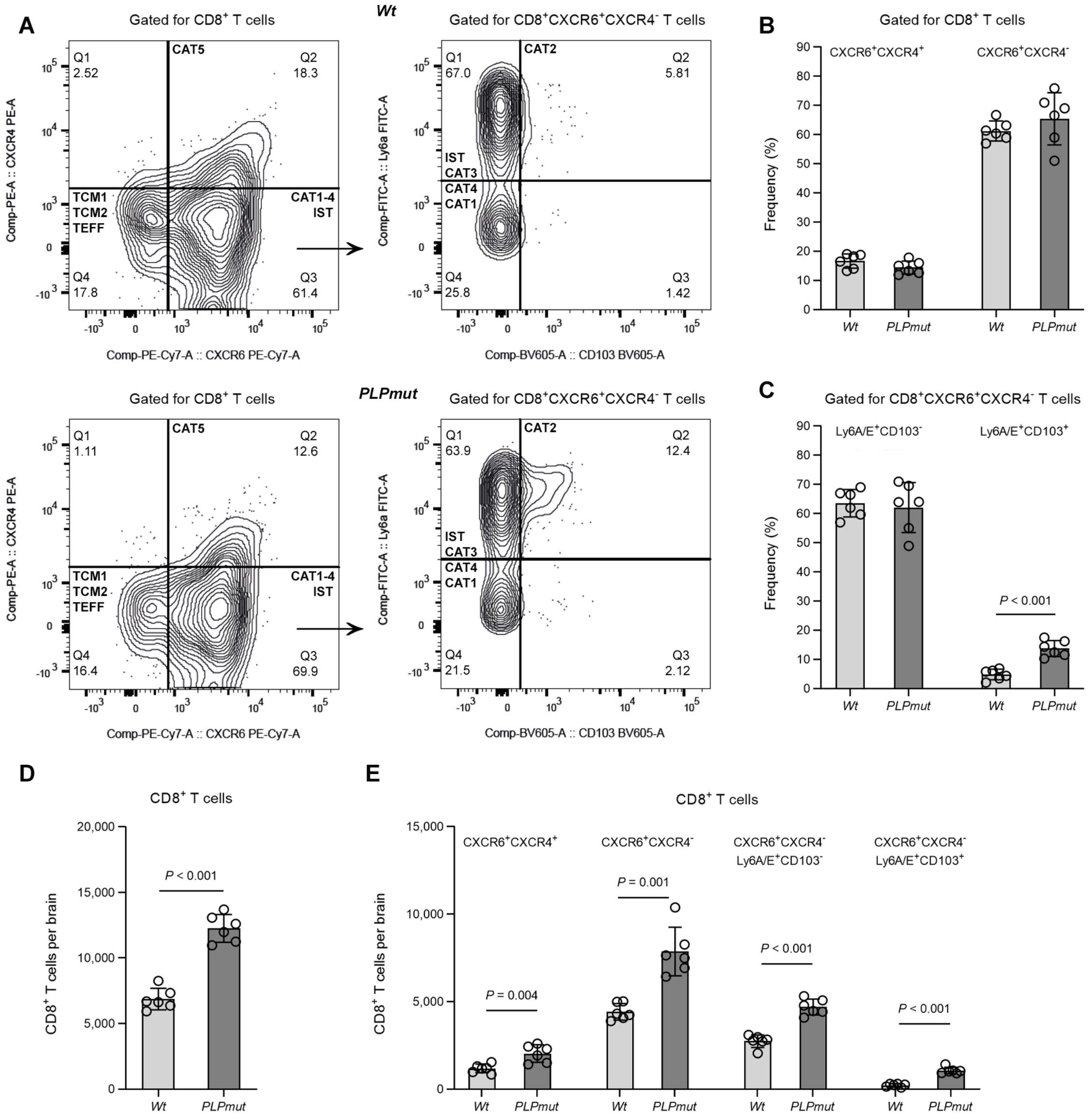

Figure S1. Flow cytometry validates the heterogeneity and accumulation of CD8⁺ T lymphocyte subsets in the myelin mutant CNS, related to Figure 1

(A) Representative plots of flow cytometric analysis of single, viable $CD45^{high}CD8^+$ T cells freshly isolated from brains of 15-month-old *Wt* (top) and *PLPmut* (bottom) mice. Gated $CD8^+$ T cells are analyzed for expression of CXCR4, CXCR6, Ly6A/E, and CD103. Percentages and identity of the respective cells are indicated in the quadrants. (B) $CD8^+$ T cells comprise similar proportions of $CXCR6^+CXCR4^+$ cells (CAT5) and $CXCR6^+CXCR4^-$ cells (CAT1-4, IST) when comparing *Wt* and *PLPmut* mice (n = 6 mice per group). (C) Among $CD8^+CXCR6^+CXCR4^-$ T cells, $Ly6A/E^+CD103^+$ cells (CAT2) but not $Ly6A/E^+CD103^-$ cells show an increased frequency. (D). Total numbers of $CD45^{high}CD8^+$ T cells and (E) the different subsets per brain reflect a significant accumulation of most populations with a disproportionally increased number of CAT2 cells. B-E: unpaired Student's t-test. Data are presented as the mean ± SD. All data represent at least three independent experiments.

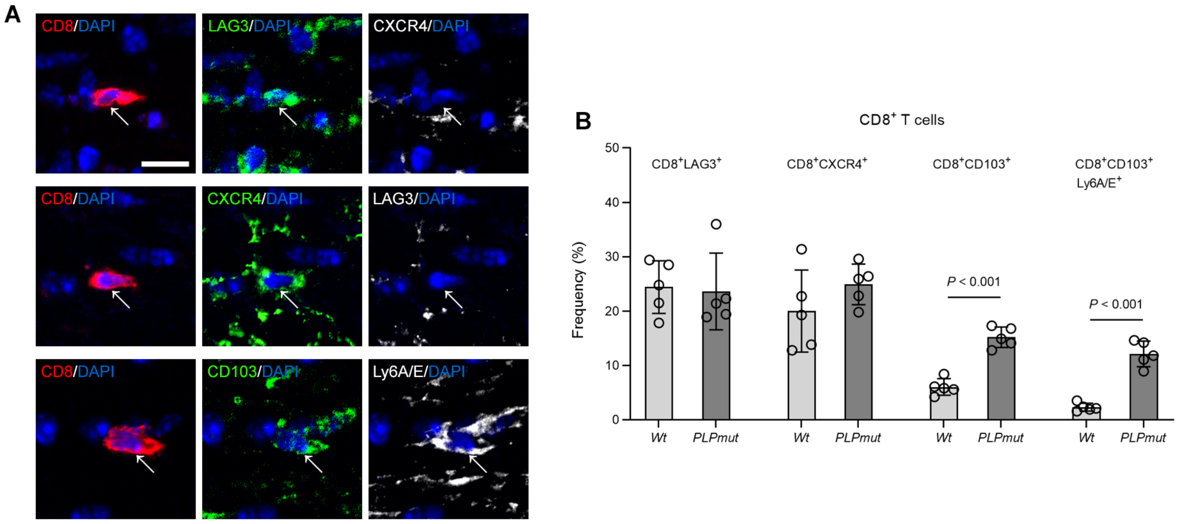

Figure S2. Immunohistochemistry validates the heterogeneity and activation of $CD8^+$ T cell subsets in the myelin mutant CNS, related to Figure 1

(A) Immunofluorescence detection of CD8, LAG3, and CXCR4 or CD8, CD103, and Ly6A/E in the optic nerves of 9-month-old *Wt* and *PLPmut* mice. $CD8^+$ T cells (arrows) show heterogenous expression of these markers. Scale bar, 10 µm. (B) Quantification of $LAG3^+$ (CAT1), $CXCR4^+$ (CAT5), $CD103^+$ (CAT2) subsets among $CD8^+$ T cells as well as Ly6A/E immunoreactivity of CAT2 (n = 5 mice per group). There is an increased frequency of $CD103^+$ cells with increased Ly6A/E expression detectable among $CD8^+$ T cells in *PLPmut* mice. B: unpaired Student's t-test. Data are presented as the mean ± SD. All data represent at least three independent experiments.

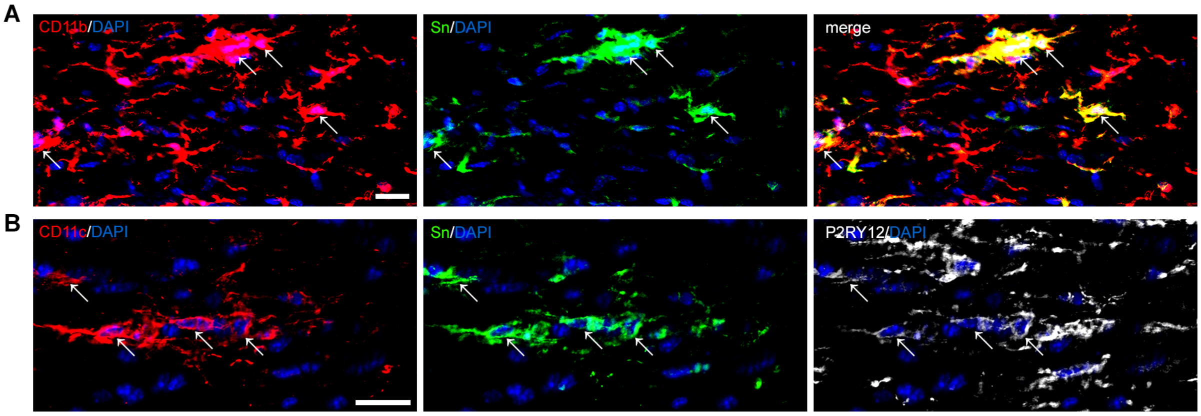

Figure S3. Expression of Sn and CD11c identifies activated microglia in *PLPmut* mice, related to Figure 3

(A) Representative immunofluorescent detection of Sn on CD11b$^+$ microglia in the optic nerves from 15-month-old *PLPmut* mice. Arrows indicate Sn$^+$ cells. Quantifications are provided in Fig. 3 G. Scale bar, 20 μm. (B) Representative immunofluorescent detection of CD11c, Sn, and P2RY12 in the optic nerves from 15-month-old *PLPmut* mice. Arrows indicate CD11c$^+$Sn$^+$P2RY12$^+$ cells. Sn and CD11c expression is restricted to activated microglia with reduced P2RY12 expression. Scale bar, 20 μm. All data represent at least three independent experiments.

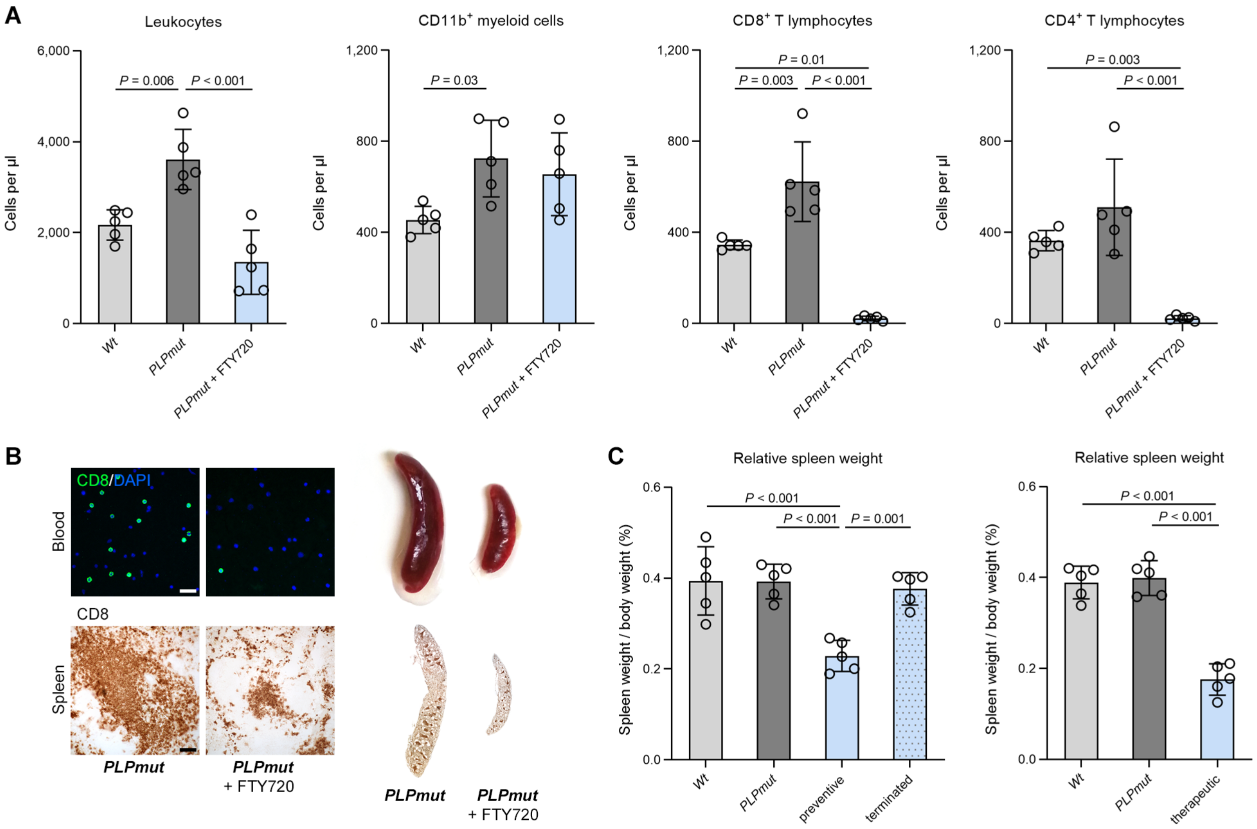

Figure S4. Sphingosine-1-receptor modulation with FTY720 depletes circulating T cells in *PLPmut* mice, related to Figure 3
(A) Flow cytometric quantification of leukocytes, CD11b$^+$ myeloid cells, CD8$^+$ T cells, and CD4$^+$ T cells per µl blood from *Wt*, *PLPmut* and FTY720-treated *PLPmut* mice (n = 5 mice per group) after the preventive regimen shown in Fig. 3 B. (B) Representative immunohistochemical detection of CD8$^+$ T cells in blood and spleen of *PLPmut* and FTY720-treated *PLPmut* mice. Scale bars, 20 µm (top) and 40 µm (bottom). Numbers of CD8$^+$ T cells and spleen volume are strongly decreased by FY720. (C) Analysis of the relative spleen weights in *Wt*, *PLPmut* and FTY720-treated *PLPmut* mice (n = 5 mice per group) using regimens indicated in Fig. 3 B. Preventive FTY720 treatment reduces spleen weight in *PLPmut* mice which is restored after termination at half time. Therapeutic FTY720 treatment also reduces spleen weight. C: one-way ANOVA with Tukey's multiple comparisons test. Data are presented as the mean ± SD. All data represent at least three independent experiments.

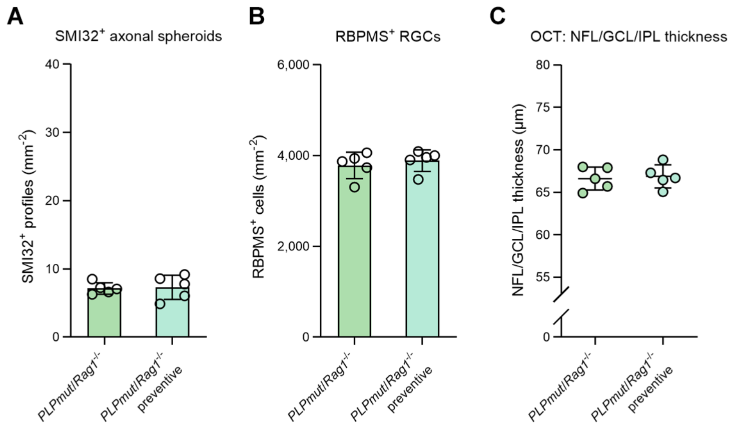

Figure S5. Beneficial effects of FTY720 treatment in *PLPmut* mice are immunomodulatory, related to Figures 3 and 4

(A) Quantification of SMI32$^+$ axonal spheroids in the optic nerves, (B) RGCs, and (G) OCT analysis of the innermost retinal composite layer (NFL/GCL/IPL) in peripapillary circle scans in 9-month-old *PLPmut*/*Rag1*$^{-/-}$ mice with or without preventive FTY720 treatment (n = 5 mice per group). FTY720 treatment has no beneficial effect on the mild neurodegeneration observed in immunodeficient *PLPmut* mice. A-G: unpaired Student's t-test. All data represent at least three independent experiments.

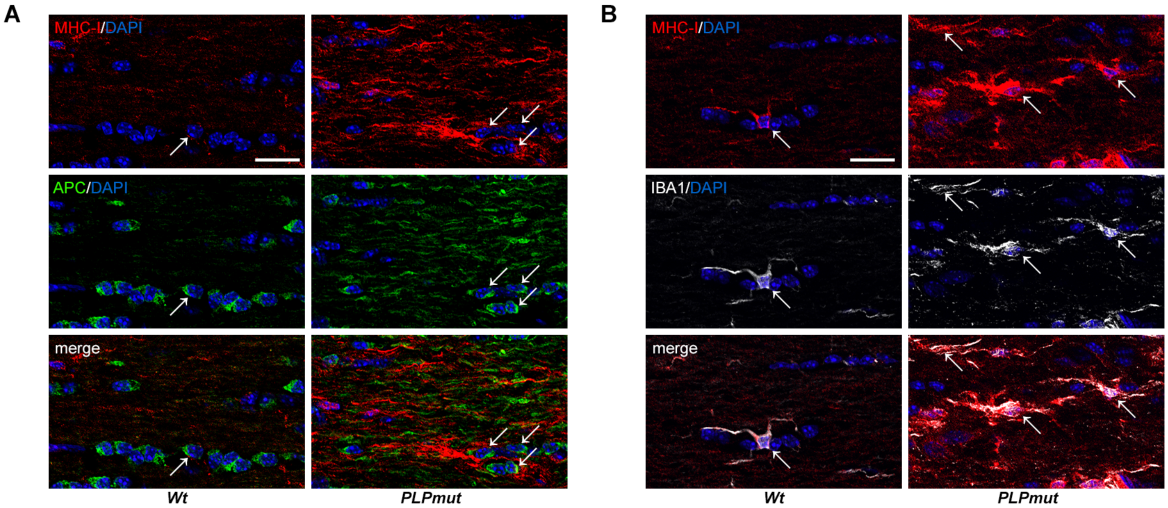

Figure S6. Increased expression of MHC-I on oligodendrocytes and microglia in *PLPmut* mice, related to Figure 7
(A) Representative immunofluorescent detection of MHC-I on APC$^+$ oligodendrocytes or (B) IBA1$^+$ microglia in the optic nerves from 18-month-old *Wt* and *PLPmut* mice. Arrows indicate MHC-I$^+$ cells. Quantifications are provided in Fig. 7 H and I. Scale bars, 20 µm. All data represent at least three independent experiments.
